# Opportunistic fungi in tropical beaches: Diversity, WHO fungal priority pathogens, and antifungal resistance case study

**DOI:** 10.1038/s41598-026-55443-7

**Published:** 2026-06-04

**Authors:** Syahriar Nur Maulana Malik Ibrahim, Chayaporn Lakmuang, Teeratat Kaewjon, Panwad Tongchai, Gregorius Nico Adi Setiawan, Phetcharin Hinmo, Ariya Chindamporn, Nattapol Kraisitudomsook, Nuttapon Pombubpa

**Affiliations:** 1 Department of Microbiology, Faculty of Science, Chulalongkorn University, Bangkok, 10330 Thailand; 2 Department of Microbiology, Faculty of Science, Southeast Asia Antifungal Resistance Monitoring Initiative (SEA-ARMi), Chulalongkorn University, Bangkok, 10330 Thailand; 3 –Bioinformatics and Computational Biology Program, Graduate School, Chulalongkorn University, Bangkok, 10330 Thailand; 4 Department of Microbiology, Faculty of Medicine, Chulalongkorn University, Bangkok, 10330 Thailand; 5 Department of Microbiology, Faculty of Science, Mahidol University, Bangkok, 10400 Thailand

**Keywords:** WHO FPPL, Opportunistic pathogens, Diversity, Antifungal resistance, Fungal community structure, Beach reservoirs, Microbiology, Microbial ecology

## Abstract

Tropical coastal regions, despite being major tourist destinations, remain underexplored in terms of their role as potential reservoirs of opportunistic fungal pathogens, particularly those listed on the WHO Fungal Priority Pathogens List (FPPL). This case study presents the first integrative molecular and culture-based assessment of fungal communities in beach sand from three sites along the coastline of Prachuap Khiri Khan, Thailand. Using ITS metabarcoding and culture-based methods, we characterized fungal diversity, detected taxa from genera that include FPPL-listed species, and assessed antifungal susceptibility. The results indicated differences in beta diversity among sites; environmental variables showed directional influence on community variation, with temperature significantly associated with the observed patterns, while moisture was not statistically significant. The urban adjacent SON site exhibited a higher prevalence of FUNGuild predicted opportunistic pathogens, while the more remote DSN site showed fewer. Five of the thirteen isolated fungi exhibited elevated minimum inhibitory concentrations (MICs) against azoles and flucytosine, isolates affiliated with *Aspergillus* section *Terrei*, which also showed genetic similarity to clinical strains. These findings suggest that recreational beaches may act as potential environmental reservoirs of opportunistic and antifungal-resistant fungi. However, only *Fusarium neocosmosporiellum* TPS1-10, a high-priority FPPL pathogen, was successfully isolated among taxa detected by ITS metabarcoding, and it did not exhibit elevated MIC values. Overall, these results highlight the need for routine fungal monitoring in recreational beach environments, in accordance with WHO guidelines, particularly in the context of increasing antifungal use, climate change, and growing beach tourism.

**Supplementary Information:**

The online version contains supplementary material available at 10.1038/s41598-026-55443-7.

## Introduction

During the past few years, mycologists have been voicing concerns about the outbreaks of fungal pathogens that are taking place across the globe^1^. Fungal pathogens have killed more than 1.5 million people annually^2^. Recently, there has been growing attention to fungi in public health policymaking, highlighted in 2022 when the World Health Organization (WHO) issued a fungal priority pathogens list (FPPL)^3^. This underscores the urgent need to enhance surveillance, address knowledge deficiencies, and bolster public health measures for the listed fungal pathogens. According to this list, the critical priority of fungal pathogens are *Cryptococcus neoformans*, *Candida auris*, *Candida albicans*, and *Aspergillus fumigatus*. These pathogens are concerning due to high mortality rates and limited resistance surveillance data^3^. All four pathogens have been found in Thailand and caused a significant burden in hospitals nationwide^4^. However, the concern for investigating natural reservoirs of WHO FPPL in Thailand remains low, highlighting the need for intensified surveillance and targeted environmental monitoring to understand potential sources and transmission risks.

Between 2007 and 2025, several opportunistic fungal pathogens listed on the WHO FPPL have been reported in various Thai environments. *Fusarium* spp. were detected on indoor surfaces in flood-affected areas of Trang province^5^. Airborne sampling at Chong Chom border market (Surin province) revealed four high-priority WHO FPPL species, *Acremonium* spp., *Fusarium* spp., *Mucor* spp., and *Rhizopus* spp., from environments such as footpaths, handicraft stalls, electronics shops, and secondhand clothing vendors^6^. In Bangkok and Songkhla, air pollution samples carried WHO FPPL genera, *Fusarium*, *Candida*, *Curvularia*, and *Cryptococcus*, though further species-level identification is needed^7^. Hospitals in Northeast Thailand frequently report infections by WHO FPPL fungi such as *C. albicans* and *Talaromyces marneffei*^8,9^. Soil sampling across 23 provinces identified *Scedosporium* spp. in tourism parks and pigsty farm drainage sites, contributing to national monitoring efforts for scedosporiosis^10,11^. In agricultural settings, *A. fumigatus* was also detected in rice field soils^12^. Across 18 northeastern provinces, WHO FPPL genera including *Candida tropicalis*, *Acremonium* spp., *Fusarium* spp., *Rhizopus* spp., *Scedosporium* spp., *Mucor circinelloides*, *T. marneffei*, *Curvularia lunata*, *Falciformispora senegalensis*, and *Lichtheimia* sp. have been documented^13^. In aquatic systems, *A. fumigatus* was isolated from sediments in the Gulf of Thailand and Andaman Sea^14^, while *C. albicans*, *C. tropicalis*, and *Candida krusei* were recovered from mangroves and peat swamps in seven provinces^15,16^. Coastal beaches, including Kung Wiman Beach, were found to harbor *Fusarium* spp. in sand and plastic debris^17^. Additionally, marine sponges from Samae-san, Mu, and Khram Islands yielded yeasts such as *C. tropicalis* and *Candida parapsilosis*^18^. Yet, there is no recorded study focusing on opportunistic fungal pathogens and WHO FPPL in Thai coastal areas, despite their popularity as tourist destinations and potential as hotspots for fungal outbreaks.

Thailand, located in Southeast Asia, is geographically divided into six natural regions based on its shape and geology: North region with the plant fields, mountains and forests; The central region with enormous plant field, city and industry; The northeast region with plateau and semi-arid farm; The east region with fields and coastal peninsula area; The western and south regions with tropical islands and a lengthy coastline of the peninsula^19,20^. From the territorial division, the central and south region of Thailand have a beach area. Three distinct types of beaches are found in Thailand: stone, muddy, and sandy beaches, where the sand beaches are predominantly located along the western Gulf of Thailand, with Prachuap Khiri Khan hosting one such example (https://km.dmcr.go.th/c_56/s_77/d_2761*).* The Ministry of Natural Resources and Environment report in 2017, this province boasts a total of 40 recreational beaches, including Thap Sakae, Son, and Don Samran (https://km.dmcr.go.th/c_53*).* Thap Sakae Beach is among the recommended locations to visit for viewing the evening sunset (https://travel.trueid.net/detail/r5DEDgN9Q2ao*)*, Son Beach is situated within an urban environment, with trees (*Casuarina equisetifolia*) partially shading the area (https://beachsearcher.com/en/beach/764201551/hat-son-beach*)*, and Don Samran Beach is nearly deserted beach even at the peak of the season (https://beachsearcher.com/en/beach/764201555/don-samran-beach*).* Given the adaptability of fungal communities to diverse environments, the presence of opportunistic fungal pathogens, especially WHO FPPL, in coastal areas, especially popular tourist sites, could emerge. Furthermore, widespread use of fungicides, particularly azoles, has led to contamination of coastal zones in Thailand Gulf^21^. This raises critical public health concerns, including the potential emergence of antifungal-resistant fungal strains in coastal ecosystems.

Among the WHO FPPL, *Candida auris* has emerged as a critical global health concern due to its antifungal resistance and high mortality rate, reaching up to 40%^22^. Notably, *C. auris* has been isolated from the Andaman Sea^23^ and coastal regions of Colombia^24^, both within the tropical zone. This geographic similarity suggests the potential for *C. auris* and other WHO FPPL to occur in Thailand’s coastal areas, particularly in Prachuap Khiri Khan province, located at a similar latitude (11.0–11.5°N). Previous studies indicate that sand and seawater may serve as hidden reservoirs for pathogenic fungi^25,26^. Despite this, data on the presence of such pathogens in Thai coastal environments, especially in high-traffic recreational sites like the beaches of Prachuap Khiri Khan, remain scarce. With an average of 6.5 million visitors between 2019 and 2021^27^, these beaches may represent potential environmental reservoirs of fungal pathogens and possible sources of exposure, warranting targeted investigation.

Fungal diversity in environmental samples has traditionally been assessed using conventional mycological techniques, such as fungal isolation onto medium or direct site observation^28^. However, these methods are limited in their ability to detect unculturable or low-abundance species^29^. The advent of High-Throughput Sequencing (HTS), also known as Next Generation Sequencing (NGS), has revolutionized microbiology by providing deeper insights into fungal communities and their ecological roles—including decomposition, nutrient cycling, and pathogenic interactions^30–32^. Moreover, phylogeographic analyses can be done from molecular sequencing data. It can provide valuable insights into intraspecific relationships, offering a deeper understanding of the population structure and distribution of fungal strains, including antifungal-resistant variants, from both environmental and clinical sources^33^. This approach is crucial for identifying potential sources of fungal pathogen outbreaks. This can be used as an approach to enhance the detection of antifungal resistant opportunistic fungal pathogens, while enabling investigation of their geographic distribution pattern.

Moreover, due to the impact of climate change, the emergence of opportunistic fungal pathogens, including strains potentially resistant to antifungal treatments, is expected to rise in the future^1^. This risk may be exacerbated by antifungal pollution along Thailand’s Gulf coastal beaches^21^. By integrating culture-based techniques with ITS metabarcoding via NGS, along with antifungal susceptibility testing, this study aims to raise awareness among healthcare professionals and the public about the potential health risks posed by coastal fungal pathogens. Additionally, this is the first phylogeographic study of antifungal-resistant fungal strains isolated from beach sand in Thailand. The study focuses on characterizing fungal communities in Prachuap Khiri Khan coastline, specifically opportunistic pathogens and WHO FPPL, as well as antifungal susceptibility and regional dispersal of resistant strains. We hypothesis that: (1) Environmental conditions at each beach influence the composition of fungal communities, particularly opportunistic pathogens; (2) ITS metabarcoding and culture-based methods provide complementary insights into the detection of these pathogens; (3) Beach environments may harbor fungi with antifungal resistance; and (4) Antifungal-resistant strains found in beach sand may be genetically related to clinical strains. The findings from this study could offer valuable guidance for detecting and mitigating future outbreaks involving WHO FPPL in Thailand. It also contributes to the research recommendations from WHO guidelines on recreational water quality in coastal, specifically for opportunistic fungal pathogens^34^.

## Results

### Environmental factors affect the fungal community

**Fig. 1:**
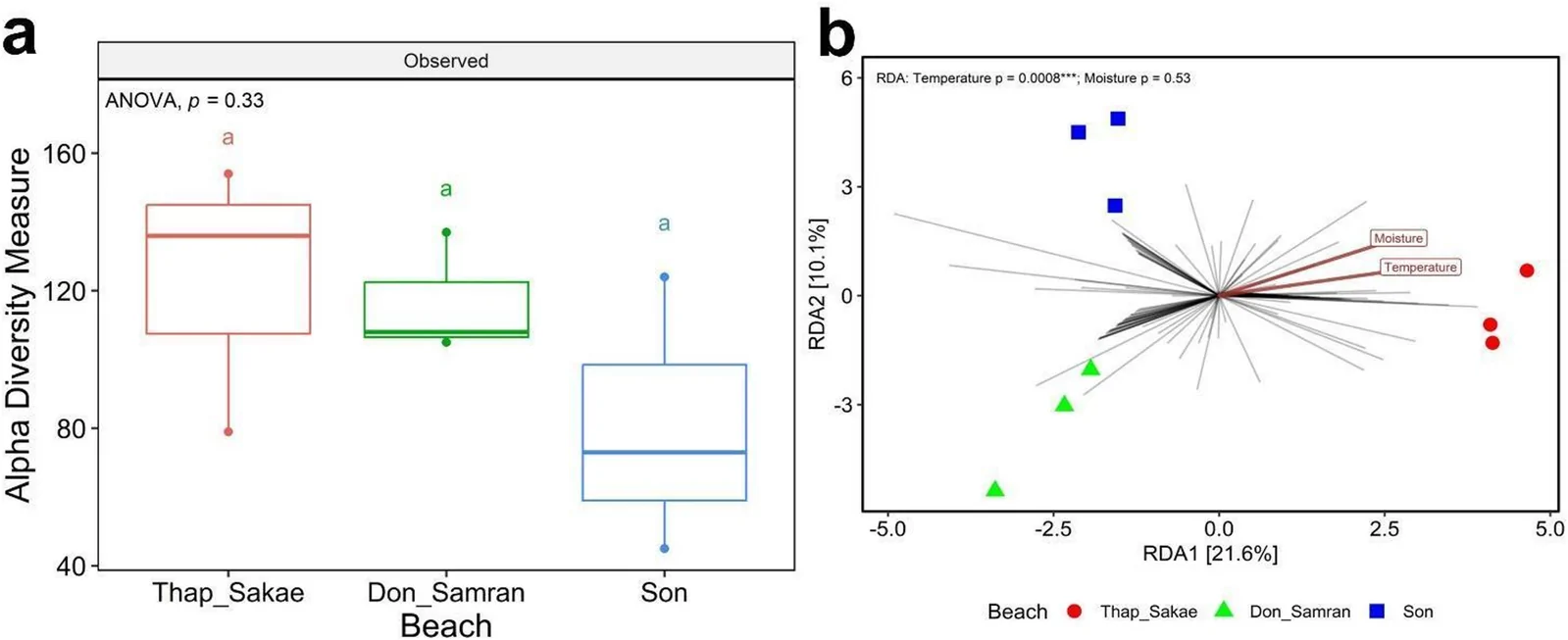
(**a**) Alpha diversity observed of the richness. Boxplots show 25th and 75th percentile while median was shown as lines inside boxes. Tukey HSD not significant differences (*p* > 0.05) are indicated by similar letters. (**b**) Beta diversity RDA ordination of fungal community composition across three beaches. Environmental variables significantly explained community variation (R² = 0.316, *p* = 0.008), with temperature significant and moisture not. Points are colored by beach: TPS (red), DSN (green), and SON (blue).

From nine samples collected across three beaches, the demultiplexed ITS sequence ranged from 18,565 to 57,690. The alpha diversity results showed that observed amplicon sequence variants (ASVs) richness did not significantly differ among the three locations (*p* = 0.33) (Fig. 1a). Beta diversity was analyzed using Principal Component Analysis (PCA), Principal Coordinates Analysis (PCoA), and Redundancy analysis (RDA). The PCA plot (Figure S1a) illustrates associations between dominant genera and sampling beaches, with selected abundant genera from the three locations highlighted for comparison. The class Agaricomycetes was consistently present across all beaches. Specifically, four genera—*Nigrospora*,* Arachnion*,* Coprinellus*, and *Candida*—were found exclusively in the TPS sand sample, while *Letendraea* was identified in both DSN and SON beaches The PCoA plot showed that fungal community composition differed among beaches (PERMANOVA, R² = 0.383, *p* = 0.005) (Figure S1b). However, homogeneity of multivariate dispersion was not met (betadisper *p* = 0.0366) (Figure S1c), indicating that differences may be partially influenced by unequal dispersion among groups and should be interpreted with caution. RDA based on CLR-transformed genus-level data showed that the overall model was significant (R² = 0.316, adj. R² = 0.088, *p* = 0.008), indicating that environmental variables explained a moderate proportion of variation, although the adjusted R² suggests limited explanatory power after accounting for sample size and model complexity. Among the environmental variables tested, temperature had a significant effect (*p* = 0.0008), whereas moisture was not significant (*p* = 0.53). The RDA plot (Fig. 1b) showed moderate separation among beach sites, with TPS (temperature 35.5 °C and moisture 8) displaying a distinct composition along RDA1. Due to strong collinearity, pH and sand were perfectly correlated (*r* = 1.00) and were excluded from the RDA to prevent redundancy and overfitting.

**Fig. 2:**
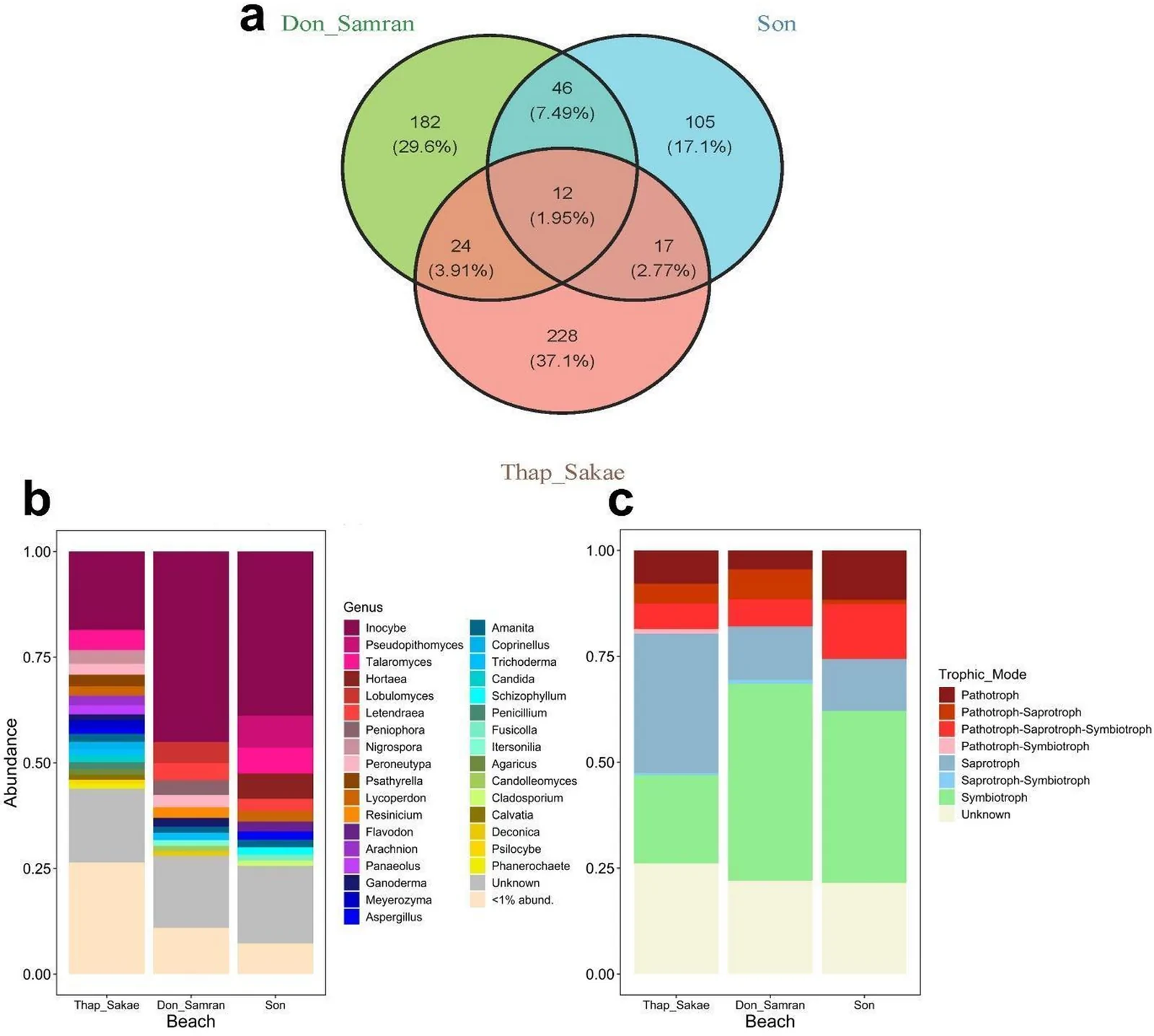
The fungal abundance and distribution from 614 ASVs of three beaches. **(a)** Total fungal distribution in the Venn Diagram, which represents the beach in red: TPS (Thap Sakae), green: DSN (Don Samran), and blue: SON (Son). **(b)** Fungal taxonomic composition based on the genus-level. Genera grouped as “<1% abund.” comprising 131 ASVs. **(c)** Fungal trophic composition based on FUNGuild from 614 ASVs, different colors indicated seven trophic modes.

A Venn diagram (Fig. 2a) was used to illustrate the distribution of fungal communities, revealing a total of 614 ASVs, with the highest proportion (37.1%) observed in the TPS samples. Further analysis identified 33 genera comprising more than 1% of the total abundance (Fig. 2b), with *Inocybe* (Basidiomycota) emerging as the dominant genus shared across all three beaches, accounting for approximately 20–45% of each community. Each site also harbored unique taxa. TPS samples included 12 genera exceeding 1% abundance, predominantly from Basidiomycota: *Psathyrella*, *Arachnion*, *Panaeolus*, *Coprinellus*, *Agaricus*, *Calvatia*, *Psilocybe*, and *Phanerochaete*; alongside four Ascomycota: *Candida*, *Meyerozyma*, *Nigrospora*, and *Penicillium*. DSN samples featured six unique genera, including four Basidiomycota (*Peniophora*, *Resinicium*, *Candolleomyces*, *Deconica*), one Ascomycota (*Peroneutypa*), and one Chytridiomycota (*Lobulomyces*), the only chytrid taxon exceeding 1% in abundance. In contrast, SON samples contained five unique genera: three Ascomycota (*Hortaea*, *Fusicolla*, *Cladosporium*) and two Basidiomycota (*Flavodon*, *Schizophyllum*). Specifically, *Aspergillus* and *Candida* were detected at more than 1% abundance in both TPS and SON, while *Talaromyces* were present across all sites but below 1% in DSN. Genera with relative abundance below 1% were grouped into an “<1% abund.” category for visualization.

Trophic mode analysis using FUNGuild (Fig. 2c) highlighted the presence of putative opportunistic pathogenic fungi across all beaches. FUNGuild analysis assigned trophic modes with varying confidence levels, including 47.72% “highly probable,” 29.80% “probable,” and 2.93% “possible” classifications. Notably, 19.54% of taxa remained unassigned, highlighting limitations in current fungal functional databases. To enhance robustness, interpretations were primarily based on “highly probable” and “probable” assignments. Pathotroph abundance ranged from 17.9% at DSN, to 19.6% at TPS, and peaked at 25.6% at SON. TPS was dominated by saprotrophs (33.4%) and supported a moderate proportion of symbiotrophs (20.8%). In contrast, DSN showed strong symbiotrophic dominance (46.5%), with lower pathotroph (17.9%) and saprotroph (13.5%) presence. SON displayed a high symbiotroph proportion (40.6%) and the lowest saprotroph representation (12.3%), coupled with the highest pathogenic load. In summary, TPS was characterized by active organic decomposition, DSN by mutualistic symbioses in a relatively undisturbed setting, and SON by elevated pathogenic potential, likely reflecting environmental stress or anthropogenic influence.

### The detected of opportunistic fungal pathogen among three beaches

**Fig. 3:**
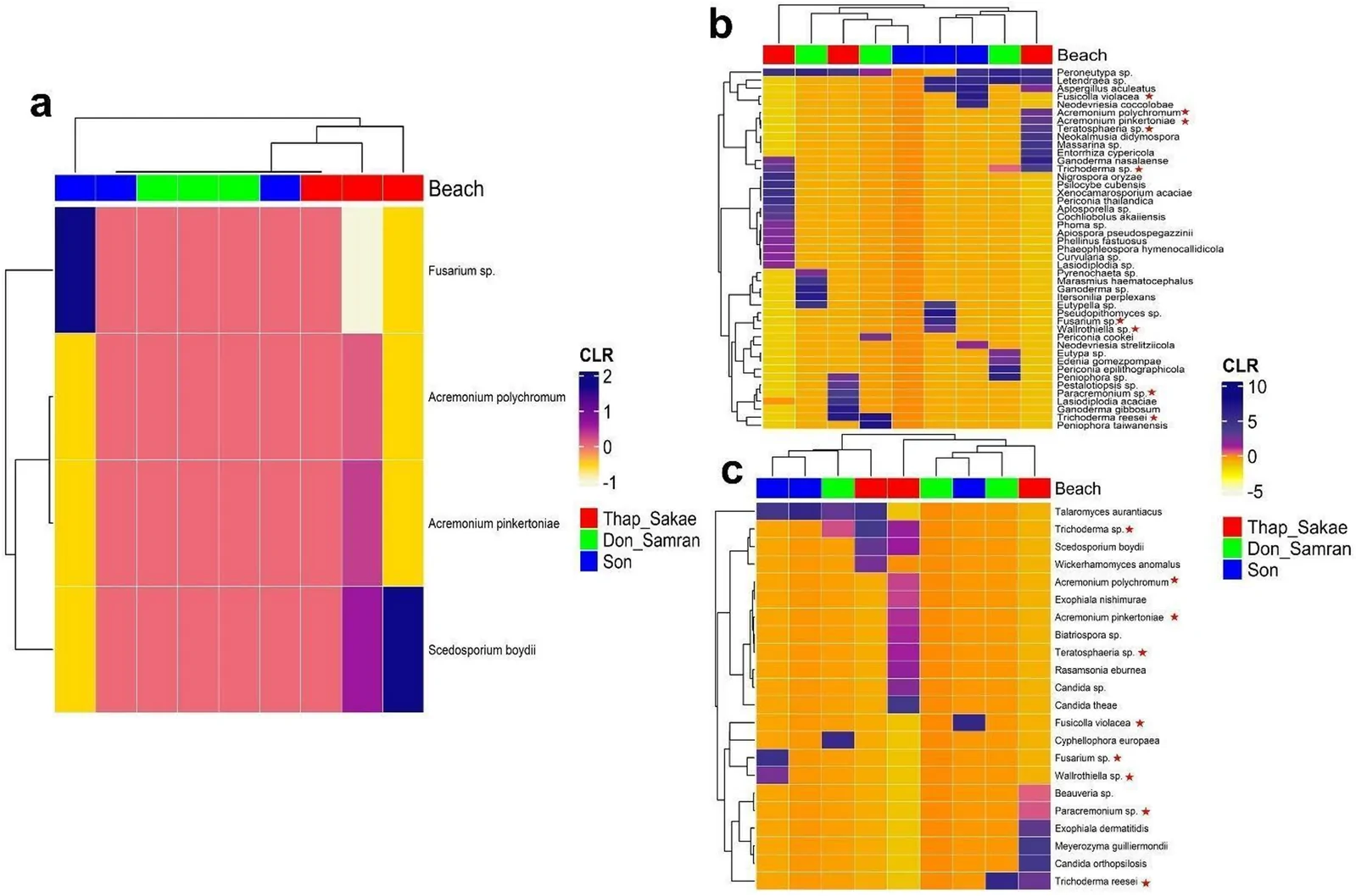
The heatmap of fungal opportunistic pathogen that was detected from three beaches: (**a**) based on WHO FPPL (Central log-ratio (CLR), −0.99 to 1.68), (**b**) plant pathogen (CLR, 1.76 to 7.78), and (**c**) animal pathogen (CLR, −1.64 to 8.11) based on FUNGuild. White color was the lowest and blue was the highest abundance based on CLR. The beach was presented in red: TPS (Thap Sakae), green: DSN (Don Samran), and blue: SON (Son). The red star (★) indicates taxa predicted to be pathogens in animals and plants. Abundance levels are represented on a color scale, where white indicates the lowest abundance and blue the highest.

The heatmap (Fig. 3a) portrayed the relative abundance of four opportunistic fungal species taxa identified based on the WHO FPPL across three coastal beaches. The abundance pattern shows that *Fusarium* sp. is widely distributed across all sampling sites, with the highest abundance observed at SON and slightly lower levels at TPS. In contrast, *Scedosporium boydii* exhibits a more localized distribution, with notable abundance primarily at TPS. Meanwhile, *Acremonium polychromum* and *A. pinkertoniae* display relatively consistent presence across all sites. At DSN, the fungal community showed lower diversity in terms of WHO FPPL-related species, with moderate and uniform levels of the *Acremonium* spp., *Fusarium* sp. and *S. boydii*. This suggests that DSN may harbor a less clinically significant fungal profile compared to TPS and SON, potentially due to variations in environmental conditions, human activity, or pollution levels.

Additionally, a total of 58 taxa were predicted to be opportunistic pathogens based on FUNGuild (Table S1), including 36 plant pathogens, 13 animal pathogens, and 9 taxa with the potential to infect both plants and animals (indicated by a red star). These taxa were analyzed using the heatmap in Fig. 3b for the plant pathogen and Fig. 3c for animal pathogen. Nine taxa predicted to be both plant and animal pathogens included *Acremonium pinkertoniae*, *Acremonium polychromum*, *Fusarium* sp., *Fusicolla violacea*, *Paracremonium* sp., *Teratosphaeria* sp., *Trichoderma reesei*, *Trichoderma* sp., and *Wallrothiella* sp. Most of these opportunistic pathogens were found in TPS samples, which contained 31 of the 45 plant pathogens and 18 of the 22 animal pathogens. DSN samples had the second highest abundance, with 14 plant pathogens and 4 animal pathogens, followed by SON samples, which contained 10 plant pathogens and 4 animal pathogens. The shared plant pathogen taxa across the three beaches included *Peroneutypa* sp. and *Letendraea* sp., while *Talaromyces aurantiacus* was the only shared taxon of animal pathogens. The taxa shared between TPS and DSN included *Trichoderma* spp., which are predicted to be both plant and animal pathogens. To further confirm the species and their viability within the WHO FPPL, we proceeded to isolate the fungi and test their antifungal susceptibility.

Of the thirteen isolates, nine were identified to the genus level, matching the results of ITS metabarcoding identification (Table S2), including five *Aspergillus*, two *Trichoderma*, and two *Montagnula*. Specifically, as shown in Table 1. All *Aspergillus* isolates were obtained from TPS at incubation temperatures of 25 °C and 35 °C. Isolates no. 1 and 2 were affiliated with *Aspergillus terreus* based on ITS and beta tubulin sequences from the 35 °C incubation. Three *Trichoderma* were also obtained from TPS, but only at the 25 °C incubation. In contrast, only three fungi were isolated from SON, all of which had less than 90% identification similarity in the database. These included *Caliciopsis* sp. SON3-8 from the 35 °C incubation and two *Montagnula* isolates (SON3-13 and SON3-17) from the 25 °C incubation. For the remaining isolates (isolates 3 and 6–8), discrepancies between culture-based and ITS metabarcoding identification were observed. These mismatches were categorized into two types: (i) absence of corresponding sequences in the metabarcoding dataset (“no detection”), and (ii) detection at a different sampling site than the original isolate (“site mismatch”). Additionally, isolates with sequence identity below 90% were flagged with an asterisk, indicating low-confidence taxonomic assignment. Overall, these discrepancies highlight methodological differences between culture-based isolation and metabarcoding approaches, particularly in detection sensitivity and spatial representation.

**Table 1:** The validation results from identification based on culture-based identification to ITS metabarcoding identification.

Isolate no.	Sampling Site	Temperature Incubation (ºC)	Percent Identity (%)		Identification Result
			ITS	Beta-tubulin	
1	TPS1	35	97	99.63	*Aspergillus terreus*
2	TPS1	35	97	96.85	*Aspergillus terreus*
3	TPS1	35	92	82.66	*Acrophialophora levis* ^a^
4	TPS3	35	100	98.52	*Aspergilus* sp.
5	TPS3	35	99.58	98.51	*Aspergillus laciniosus*
6	TPS2	35	87.73*	79.24*	*Corynascella* sp. ^a^
7	SON3	35	88.44*	83.02*	*Caliciopsis* sp. ^a^
8	TPS1	25	99.41	99.11	*Fusarium neocosmosporiellum* ^b^
9	TPS1	25	100	95.80	*Trichoderma* sp.
10	TPS1	25	100	100	*Aspergillus tubingensis*
11	SON3	25	99.44	87.64*	*Montagnula* sp.
12	TPS2	25	100	95.93	*Trichoderma* sp.
13	SON3	25	99.62	87.64*	*Montagnula* sp.

Asterisk (*) indicates sequence identity < 90%.

^a^ No corresponding sequence detected in the ITS metabarcoding dataset.

^b^ Sequence detected in the metabarcoding dataset but assigned to a different sampling site.

### The isolated fungi showed potential resistance to antifungal

Among the 13 fungal isolates subjected to antifungal susceptibility testing (AFST) (Table 2**)**, five isolates exhibited elevated MIC values for one or more antifungal agents. The isolate with the highest MIC values was affiliated with *Aspergillus* section *Terrei* (TPS1-2), which showed elevated MICs to five antifungals: flucytosine (> 64 µg/mL), anidulafungin (> 8 µg/mL), caspofungin (> 8 µg/mL), fluconazole (> 256 µg/mL), and micafungin (> 8 µg/mL). Two isolates exhibited moderate MICs: the *A. terreus*-affiliated isolate TPS1-1 (anidulafungin > 8 µg/mL and itraconazole > 16 µg/mL) and *Trichoderma* sp. TPS2-15 (fluconazole > 256 µg/mL and voriconazole > 8 µg/mL). Additionally, two isolates displayed high MICs for specific antifungals: *Aspergillus* sp. TPS3-4 for fluconazole (> 256 µg/mL) and *Trichoderma* sp. TPS1-11 for flucytosine (> 64 µg/mL).

**Table 2:** Anti-fungal susceptibility test of thirteen isolates using broth microdilution technique.

Sampling site	Isolate	5-FC	Amp	Ani	Cas	Flu	Itr	Mic	Pos	Vor
TPS1	*Aspergillus terreus* TPS1-1*	0.5	2	> 8*	0.03	64	> 16*	0.015	0.015	0.12
TPS1	*Aspergillus terreus* TPS1-2*	> 64*	4	> 8*	> 8*	> 256*	0.06	> 8*	6	0.5
TPS1	*Acrophialophora levis* TPS3-3	0.12	1	0.5	0.5	0.25	0.25	0.5	0.12	2
TPS3	*Aspergilus* sp. TPS3-4*	16	0.5	0.03	0.03	> 256*	0.06	0.015	0.06	0.12
TPS3	*Aspergillus laciniosus* TPS3-5	64	1	0.25	0.12	0.5	0.12	0.25	0.06	1
TPS2	*Corynascella* sp. TPS2-6	64	1	0.5	0.12	1	0.12	0.12	0.12	0.06
SON3	*Caliciopsis* sp. SON3-8	≤ 0.06	2	0.12	0.25	2	0.12	0.03	0.12	0.25
TPS1	*Fusarium neocosmosporiellum* TPS1-10	0.5	2	0.06	0.12	128	0.03	0.03	0.06	0.5
TPS1	*Trichoderma* sp. TPS1-11*	> 64*	2	0.12	0.03	8	0.12	≤ 0.008	0.06	0.06
TPS1	*Aspergillus tubingensis* TPS1-12	16	4	0.12	0.25	128	0.5	0.06	0.5	2
SON3	*Montagnula* sp. SON3-13	0.25	4	2	4	16	0.12	2	0.12	0.25
TPS2	*Trichoderma* sp. TPS2-15*	32	1	0.06	0.5	> 256*	4	2	2	> 8*
SON3	*Montagnula* sp. SON3-17	0.5	2	≤ 0.015	≤ 0.08	128	0.03	≤ 0.008	0.06	0.5

Note:.

Antifungal agents’ concentration in µg/mL, 5-FC = Fluorocytosine; Amp = Amphotericin B; Ani = Anidulafungin; Cas = Caspofungin; Flu = Fluconazole; Itr = Itraconazole; Mic = Micafungin; Pos = Posaconazole; Vor = Voriconazole.

*MIC values are reported descriptively. No clinical breakpoints or ECOFFs are available for several mold–drug combinations; therefore, isolates were not classified as susceptible or resistant (CLSI M38-A2).

### Relationship of *Aspergillus terreus *strain TPS with environmental and clinical strains

**Fig. 4:**
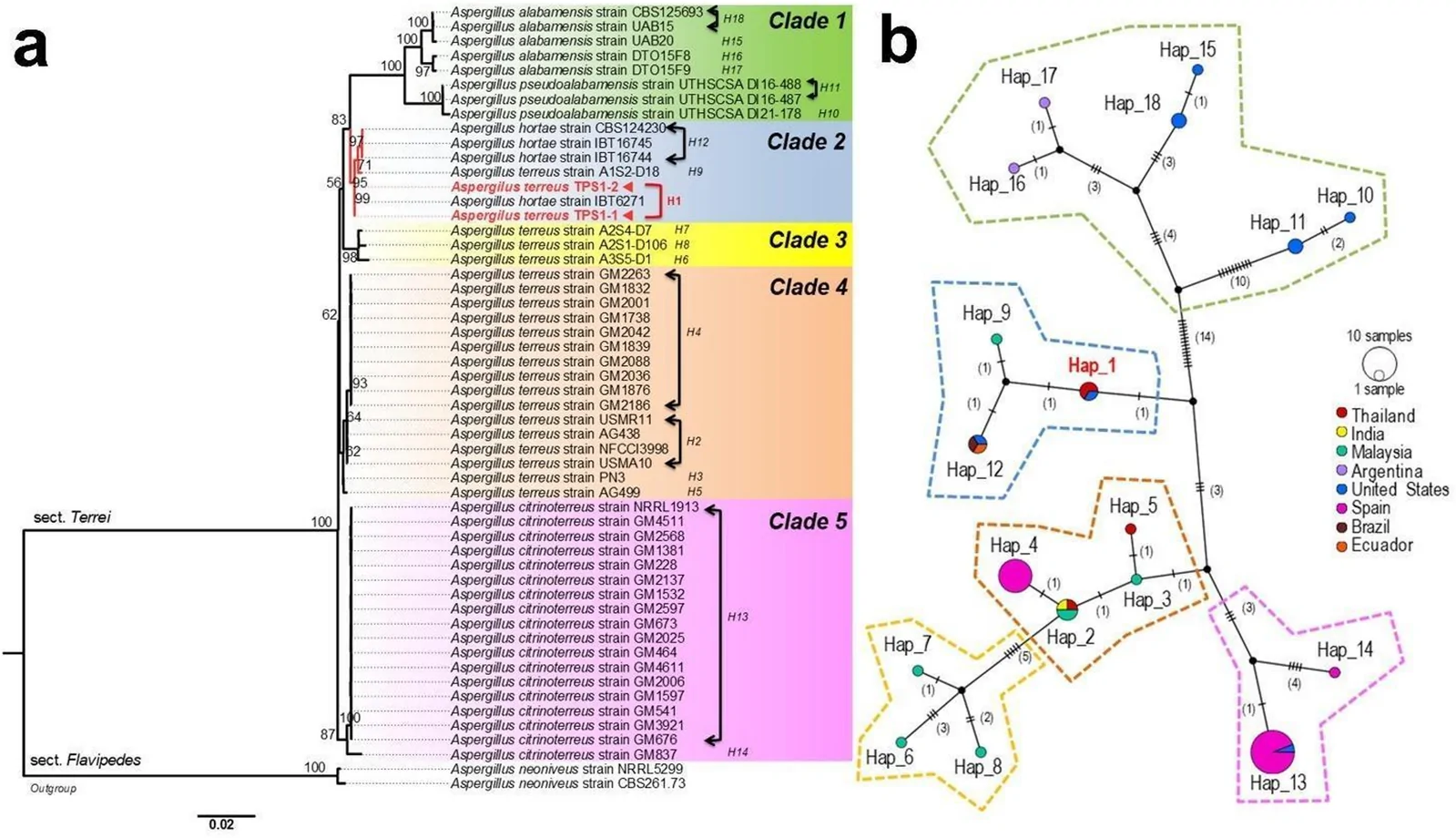
Phylogeographic analysis of *Aspergillus terreus* TPS1-1 and TPS1-2 based on concatenated ITS and β-tubulin sequences (792 bp total). (**a**) Maximum Likelihood (ML) tree constructed from 54 sequences with a genetic distance of 0.02, using *Aspergillus* section *Flavipedes* as the outgroup. The red branch highlights the clade containing *A. terreus* TPS1-1 and TPS1-2. Support values at each node are based on 1,000 ultrafast bootstrap replicates and 1,000 SH-like approximate likelihood ratio tests. (**b**) TCS haplotype network showing 18 haplotypes (Tajima’s D ≤ − 0.375, *p* = 0.625). Five clades are consistently observed in both the ML tree and the network: Clade 1 (green), Clade 2 (blue), Clade 3 (yellow), Clade 4 (orange), and Clade 5 (pink).

Phylogeographic analysis based on both the maximum likelihood (ML) phylogenetic tree and the TCS haplotype network (Fig. 4a and b) revealed distinct genetic lineages within *Aspergillus* section *Terrei*. The ML tree resolved five well-supported clades: Clade 1 comprised *Aspergillus alabamensis* and *Aspergillus pseudoalabamensis*; Clade 4 included *A. terreus* strains; and Clade 5 contained *Aspergillus citrinoterreus*. Particularly, isolates TPS1-1 and TPS1-2 from Thailand, initially identified as *A. terreus* based on ITS sequences, did not cluster within the main *terreus* clade (Clade 4). Instead, they are grouped with *Aspergillus hortae* (Clade 2), while Malaysian isolates are annotated as “*Aspergillus terreus*” forming a separate lineage (Clade 3).

The TCS haplotype network identified 18 unique haplotypes among the analyzed strains. Nucleotide diversity (π) was 0.015, indicating moderate genetic variation. A total of 58 segregating sites were found, with 45 being parsimony informative. Tajima’s D value was − 0.375 (*p* = 0.625), indicating no significant deviation from neutrality, suggesting the population is evolving under neutral conditions. The haplotype distribution provided additional insights into geographic and genetic differentiation. Thai isolates affiliated with *Aspergillus* section *Terrei* were represented by multiple haplotypes (Hap_1, Hap_2, and Hap_5), reflecting both shared and unique genetic profiles. Isolates TPS1-1 and TPS1-2 (Hap_1), initially identified as *A. terreus* based on ITS sequences, were shared with a strain from the United States, were centrally positioned in the network, suggesting a potential ancestral haplotype and possible long-distance dispersal. In contrast, *A. terreus* strain AG499 (Hap_5), found exclusively in Thailand, likely represents localized evolutionary divergence. Other notable patterns included Clade 5 (Hap_13 and Hap_14), consisting of *A.* citrinoterreus clinical strains from Spain and the United States, and Clade 4, represented by a single haplotype (Hap_4) of a clinical *A.* terreus strain from Spain. Clade 1 included *A. alabamensis* from Argentina (Hap_16 and Hap_17) and a combination of *A. alabamensis* and *A. pseudoalabamensis* from the United States, isolated from both soil (Hap_18) and clinical sources (Hap_10, Hap_11, and Hap_15).

## Discussion

Our study detected that fungal community composition, including WHO FPPL and opportunistic fungal pathogens, varies across coastal environments, highlighting the role of site-specific ecological factors. To the best of our knowledge, this is the first investigation of opportunistic fungal pathogens, including WHO FPPL, along the Prachuap Khiri Khan coastline. The detection of these potential pathogens through ITS metabarcoding, complemented by culture-based methods, raises concerns regarding potential public health risks for recreational beachgoers, particularly immuno-compromised individuals. In addition, the presence of isolates with elevated MIC values and their genetic relatedness to clinical strains suggest that coastal sands in Prachuap Khiri Khan may act as environmental reservoirs, supporting global concerns about the rise of resistant fungal strains in the context of temporal variation and pharmaceutical pollution. These findings offer valuable insights for future beach mycobiome research, particularly in relation to opportunistic fungal pathogens, as we discussed below.

### The beach environment shaped the fungal community and opportunistic pathogens

While alpha diversity (species richness) did not significantly differ among the three sampling locations (Fig. 1a), beta diversity analysis revealed distinct fungal community compositions (Figure S1a and S1b), indicating that each beach harbors a unique assemblage. This suggests that while the overall number of fungal taxa remains stable across sites, environmental factors likely play a crucial role in shaping community structure (Fig. 1b). Several fungal genera appeared to be site-specific, with *Nigrospora*,* Arachnion*,* Coprinellus*, and *Candida* exclusively found at TPS. This site specifically suggests that ecological factors, possibly combined with human activities, contribute to fungal distribution patterns. Previous research has shown that beach cleaning, mowing, and foot traffic can alter coastal ecosystems, influencing microbial diversity^34,35^. The fungal distribution highlights ecological distinctions among the beaches, with TPS exhibiting the highest proportion of unique fungal taxa (37.1%) (Fig. 2a), including 12 taxa with relative abundances exceeding 1% (Fig. 2b). This suggests that TPS offers a distinct microenvironment conducive to the growth of certain fungal species, potentially influenced by higher temperature, moisture, and anthropogenic activity (Fig. 1b, Table S3). As a more popular recreational beach than SON and DSN, increased visitor activity at TPS may contribute to the observed patterns^35^. Comparisons with previous studies on marine fungi revealed minimal overlap, with only *Dactylospora* shared between our findings and those of Suetrong et al. (2017)^36^, who studied fungi in mangrove estuarine driftwood and sand. Additionally, among the 154 marine fungi identified by Jones et al. (2006)^37^, only *Periconia* was found in both studies, reinforcing the uniqueness of potential endemic fungal communities in these coastal environments.

Environmental variation and human activity significantly influence the structure of fungal communities in coastal ecosystems, as evidenced by the distribution of fungal trophic modes (Fig. 2c). At SON, the notable presence of both pathotrophic and symbiotrophic fungi may reflect environmental stress or nutrient-poor conditions, which favor both pathogenic and mutualistic interactions. This pattern is likely associated with the site’s proximity to fishermen’s residences and local restaurants, suggesting anthropogenic contributions to fungal dispersal. The elevated abundance of pathotrophs at SON raises public health concerns, especially as this recreational beach may serve as a reservoir for potential opportunistic pathogens, including WHO FPPL. In contrast, DSN, a relatively undisturbed and less frequent beach with limited vehicular access, exhibited the highest proportion of symbiotrophic fungi. This dominance likely indicates stable ecological conditions and active mutualistic relationships, possibly with coastal vegetation, suggesting that DSN maintains a more intact and naturally functioning ecosystem. Meanwhile, TPS showed dominance of saprotrophic fungi, consistent with elevated organic input and decomposition activity. As a popular, easily accessible tourist destination, TPS likely receives increased organic matter from human presence and runoff, supporting a saprotroph-driven nutrient cycle and contributing to a moderately diverse trophic structure.

Across all three sites, unclassified trophic modes accounted for 21–26% of the fungal community, highlighting a significant gap in the functional annotation of coastal fungi, particularly in tropical regions. The presence of nearly 20% unassigned taxa underscores these limitations, and therefore trophic mode interpretations should be considered indicative rather than definitive. Additionally, the detection of several WHO FPPL genera, including *Acremonium*, *Aspergillus*, *Candida*, *Fusarium*, *Talaromyces*, and *Scedosporium*, at recreational beaches underscores potential health risks, especially for immunocompromised individuals^3^. Previous studies in Thailand have reported some of these genera, such as *Aspergillus*, *Candida*, and *Fusarium*, in coastal environments^17,18,38^, though without a focus on their pathogenicity or antifungal resistance potential. Specifically, *Talaromyces* and *Scedosporium* have not previously been reported from Thai coastal ecosystems. We underscore the need for continued fungal isolation to assess viability, coupled with molecular and ecological monitoring to improve understanding of poorly classified fungi. This is especially important in the context of climate-driven fungal emergence and the global rise in antifungal resistance.

### The potential opportunistic fungal pathogen in the beach can be detected by ITS metabarcoding and culture-based

This is the first report of the potential presence of opportunistic fungal pathogens along the recreational coastline of Prachuap Khiri Khan, Thailand, underscoring the need for further investigation due to the absence of clinical records, isolation data, and antifungal testing. WHO FPPL taxa (Fig. 3), such as *Acremonium polychromum*, *A. pinkertoniae*, *S. boydii*, and *Fusarium* sp., were exclusively detected at TPS and SON, suggesting that site-specific environmental conditions may promote their presence. TPS exhibited the highest fungal richness and recorded the highest temperature (35.5 °C) together with moisture, which may favor the growth of thermotolerant fungi^39^. Additionally, factors like higher foot traffic and organic matter deposition may contribute to the introduction and persistence of certain fungal species^35^. Higher fungal abundance in SON, potentially indicating site-specific conditions, such as salinity, nutrient load, or organic pollution, that favor its proliferation. Moreover, several detected fungal genera were potential opportunistic pathogens specific to animals (*Candida*, *Talaromyces*, *Scedosporium*), plants (*Aspergillus*), or both (*Acremonium* and *Fusarium*) (Fig. 3, Table S1). *Talaromyces aurantiacus* has been identified as an opportunistic animal pathogen, including documented infections in dogs^40^. *Scedosporium boydii* or *Pseudallescheria boydii* species complex, a medium-priority pathogen on the WHO FPPL, is considered an environmental opportunist, often linked to mycetoma infections in immunocompromised individuals^41^. *Candida orthopsilopsis* and *C. theae*, closely related to the pathogenic *C. parapsilosis* (high priority WHO FPPL), have been detected in both human and environmental samples and are known to exhibit antimicrobial resistance^42,43^. *Aspergillus aculeatus*, though primarily recognized as a plant pathogen responsible for longan fruit rot, also demonstrates antimicrobial activity against Gram-positive and Gram-negative bacteria as well as yeast-like fungi^44,45^. Among the high-priority pathogens on the WHO FPPL list are Eumycetoma causative agents (*Acremonium* spp.) and *Fusarium* spp. There are no clinical records of *A. pinkertoniae*, which has recently been reclassified as *Bulbithecium pinkertoniae*^46^. In contrast, *A. polychromum* has been isolated from both human and environmental samples^47^. *Fusarium* species are particularly notable for their production of mycotoxins, which play a key role in their pathogenicity toward both humans and plants^48^. Given these findings, increased sampling and monitoring are crucial for assessing potential health risks and evaluating the environmental distribution of fungal pathogens in the region.

The detection of genera such as *Aspergillus*, *Trichoderma*, and *Montagnula* by both ITS metabarcoding and culture-dependent methods supports the hypothesis that these approaches are complementary (Table 1 and S2). However, discrepancies between cultured isolates and metabarcoding results were observed. These mismatches may stem from limitations in public DNA reference databases, where sequences may be outdated, incomplete, or misidentified^49^. Such limitations contribute to taxonomic inconsistencies and reduce the accuracy of molecular identification. This issue is consistent with findings by Chaimusik et al. (2024)^17^, who reported dominant genera detected via ITS sequencing that were not recovered through cultivation, highlighting the selective nature of culture-based methods. Cultivation challenges were evident in this study. Despite the detection of several WHO FPPL taxa through metabarcoding, many could not be cultured. Additionally, the oligotrophic nature of beach sand provides limited nutrients, which may reduce the likelihood of successful fungal growth. Culture bias is also a known limitation, as some fungi require specific environmental conditions not replicated under standard laboratory settings. Factors such as competition for nutrients, ecological interactions, such as antagonism and necrotrophic mycoparasitism, and selective media further affect cultivation success^17,50^. Temperature emerged as a key variable influencing fungal recovery, with *A. terreus* only growing at 35 °C, indicating a thermotolerant preference, while *Trichoderma* was exclusively isolated at 25 °C, highlighting the need for species-specific incubation conditions for optimal cultivation^39^. Specifically, *A. terreus* may serve as a dominant species within the beach sand fungal community, shaping community structure through competitive and environmental interactions^51^. These findings highlight the ecological complexity of coastal fungal communities and the importance of integrating multiple methodologies to fully characterize them. The specific culture media and laboratory conditions influencing fungal isolation have also been highlighted by Chaimusik et al. (2024)^17^, who successfully isolated *Aspergillus*, *Penicillium*, *Pestalotiopsis*, *Cochliobolus*, and *Fusarium* using potato dextrose agar. Nevertheless, culture-based identification provided valuable insights into the potential relationships between taxa, complementing the ITS metabarcoding approach. These findings underscore the importance of integrating both culture-based and ITS metabarcoding methods to achieve accurate profiling of fungal communities and to better assess potential pathogenic risks. The detection of clinically significant fungal pathogens in coastal environments highlights their presence beyond clinical settings, raising public health concerns related to marine and beach-associated fungi. Based on these findings, ongoing environmental sampling and surveillance are essential for monitoring fungal occurrence and evaluating potential health risks in the region.

### Opportunistic *Aspergillus* isolates with elevated MICs and genetic relatedness to clinical strains

Isolates affiliated with *Aspergillus* section *Terrei* (putatively *A. terreus*) exhibited elevated MICs to five antifungal agents, including anidulafungin, caspofungin, micafungin, fluconazole, and flucytosine (Table 2). A similar antifungal resistance profile, characterized by high MICs for flucytosine, anidulafungin, caspofungin, fluconazole, and micafungin, was observed in *A. terreus* in an ophthalmological patient at Srinagarind Hospital, Khon Kaen^52^. *Aspergillus terreus* has been associated with azole resistance, often linked to specific alterations in the *Cyp* gene^53^. This raises concerns about treatment challenges for invasive *Aspergillus* infections, as *A. terreus* is known to have intrinsic resistance to amphotericin B and reduced susceptibility to azoles, with no previously reported resistance mechanism to echinocandins^54^. Hence, this is the first report for *A. terreus* in Prachuap khiri Khan coastline with antifungal susceptibility characterization. The high MICs observed in *Trichoderma* sp. and *Aspergillus* sp. against fluconazole and flucytosine suggest that some environmental isolates may harbor resistance mechanisms with potential clinical relevance. This is supported by Wroński et al. (2024)^55^, who detected azole-resistant fungi in seawater and sediment in Thailand, particularly in wastewater treatment facilities, rivers, and coastal areas. Based on our findings, antifungal susceptibility profiles of environmental isolates may be relevant for clinicians, particularly when managing aspergillosis cases with a history of environmental exposure or travel to this region. However, MICs were determined using the Sensititre™ YeastOne™ YO10 system, a commercial colorimetric broth microdilution assay widely used in clinical laboratories and shown to have good agreement with CLSI reference methods for yeasts and *Aspergillus* spp^56,57^. As this system is primarily optimized for yeasts, results for filamentous fungi should be interpreted as indicative rather than definitive susceptibility measures. Furthermore, in the absence of established clinical breakpoints or epidemiological cutoff values (ECOFFs) for several mold–drug combinations, MIC values are reported descriptively and should be interpreted with caution. These findings underscore the importance of routine environmental surveillance to detect and monitor potential reservoirs of fungi with elevated MICs.

The integration of ML phylogenetic tree and TCS haplotype network analyses offers valuable insights into the evolutionary dynamics and geographic distribution of *Aspergillus* section *Terrei* (Fig. 4a and **b**). The ML tree clearly delineates five clades, supporting the taxonomic separation of *A. alabamensis*, *A. pseudoalbamensis*, *A. terreus*, and *A. citrinoterreus*. Phylogenetic analysis further showed that the Thai isolates (TPS1-1 and TPS1-2), initially identified as *A. terreus*, clustered with *A. hortae* in Clade 2, while Malaysian *A. terreus* strains formed Clade 3, distinct from the main *A. terreus* clade (Clade 4). This pattern suggests possible cryptic diversity or species misidentification within the *A. terreus* complex and highlights the importance of multilocus approaches for accurate classification^49,52^.

The TCS network further supports these findings, revealing 18 haplotypes. Hap_1, shared between Thai and U.S. *A. terreus* strains and centrally positioned in the network, may represent an ancestral lineage with potential long-distance dispersal. Meanwhile, Hap_5 found exclusively in Thai *A. terreus* strain AG499 from organic fertilizer^58^, suggests localized diversification. The near-neutral Tajima’s D value (–0.375, *p* = 0.625) implies that the population is not under strong selection, supporting a stable evolutionary process^59^. Additionally, the detection of *A. terreus* in both marine environments and clinical contexts underscores its ecological versatility and clinical relevance. Given that our isolations were collected from a recreational beach, the area may represent an environmental reservoir with potential public health implications. However, this analysis was limited to ITS and β-tubulin markers, which may not provide sufficient resolution for closely related taxa within *Aspergillus* section *Terrei*. The inclusion of additional loci such as calmodulin (CaM), RPB2, or actin (act1) would enhance species-level resolution^52,60^. Overall, these findings position Thailand as a potential hotspot of genetic diversity for *Aspergillus* section *Terrei* and emphasize the need for expanded environmental and clinical surveillance to better understand the species’ dispersal patterns and pathogenic potential.

### Challenge and recommendation for the beach management

In alignment with the WHO’s Recreational Water Safety Plans (RWSP)^34^, microbiologists and mycologists can play a vital role in assessing and monitoring fungal threats in recreational coastal areas, thereby helping to mitigate potential health risks. Our study contributes critical baseline data on the diversity and clinical relevance of fungal communities along the Prachuap Khiri Khan coastline, supporting the growing recognition of marine environments as reservoirs for opportunistic and antifungal-resistant fungi. By integrating ITS metabarcoding and culture-based approaches, we provide valuable insights; however, the study has certain limitations. ITS metabarcoding, while effective for broad community profiling, may lack species-level resolution for closely related or poorly characterized taxa. Additionally, culture-based methods are inherently biased toward fungi that thrive under laboratory conditions, potentially overlooking fastidious or unculturable species. The discrepancies observed between these approaches highlight the urgent need for improved, curated fungal reference databases and the development of standardized, high-resolution methodologies for more comprehensive fungal surveillance in marine environments. Future studies, particularly those incorporating genomic and functional analyses, are essential to better understand the environmental drivers of fungal adaptation, antifungal resistance, and population dispersal. These continued efforts will be crucial in shaping public health policies and environmental management strategies, ultimately bridging the gap between environmental microbiology and clinical mycology, especially in the Asia region.

## Conclusion

This study presents the first integrated molecular and culture-based assessment of fungal communities along the Prachuap Khiri Khan coastline, revealing opportunistic and antifungal-resistant species, including WHO FPPL. Environmental factors influenced fungal composition, with TPS and SON beaches showing greater potential in clinical relevance. Notably, isolates affiliated with *Aspergillus* section *Terrei* (putatively *A. terreus*) exhibited elevated MIC values and genetic similarity to clinical strains, suggesting a potential health concern. However, species-level identification and epidemiological linkage should be interpreted with caution due to methodological limitations.Overall, these findings highlight the need for further studies and routine fungal monitoring development in recreational beaches, aligning with WHO guidelines to support early detection, targeted surveillance, and safer coastal environments.

## Methods

### Sampling collection and site

**Fig. 5:**
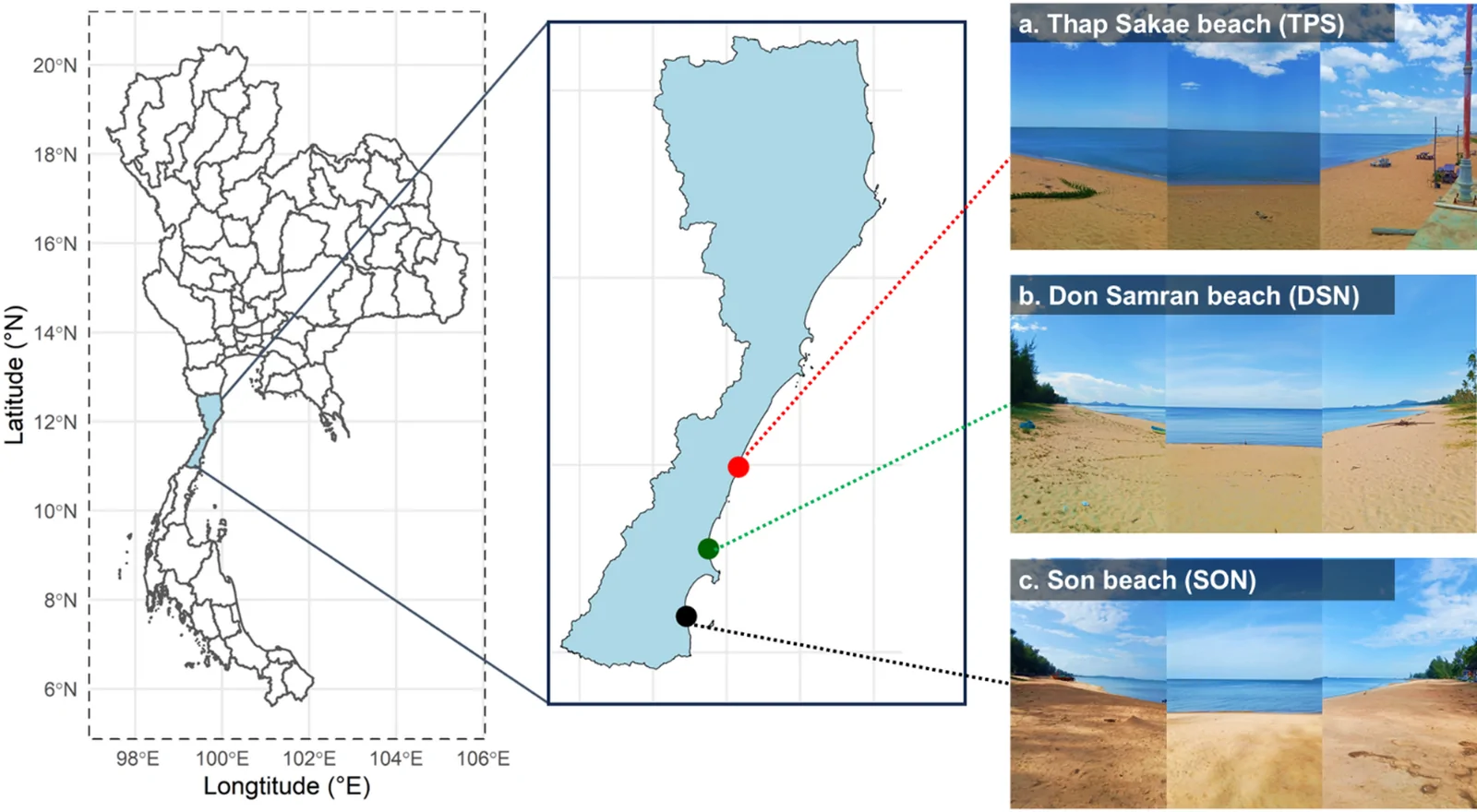
Sampling sites along the Prachuap Khiri Khan coastline, Thailand: (**a**) Thap Sakae beach (TPS), (**b**) Don Samran beach (DSN), and (**c**) Son beach (SON). Maps were generated by the authors using RStudio (v4.3.1) and edited in Microsoft Publisher. Beach photographs were taken during field sampling.

Beach sand samples were collected from 3 different beach sites (3 replicates for each beach) in Prachuap Khiri Khan including Son Beach (SON) (lat. 11°5’46”, lon. 99°29’24”), Don Samran Beach (DSN) (lat. 11°16’36”, lon. 99°33’1”), and Thap Sakae Beach (TPS) (lat. 11°29’42”, lon. 99°37’54”) on 20 August 2023. Samples were collected using systematic transect sampling along the beach shore, with a 10-meter interval between each sampling point, following the method described by Brooks (2016)^86^. The environmental properties were measured based on the moisture, sand type, temperature, and pH (Table S3). Three sample places in Prachuap Khiri Khan (Fig. 5) were selected based on the similar latitude of Corbyn’s cove beach from Arora et al., (2021)^23^, lat. 11°38’42”. Human activity was observed at TPS and SON, which both sites have restaurants and residence nearby, specifically, there are fisherman’s houses in SON beach. On the other hand, DSN beach showed low tourist visitors because the access is quite limited, especially for the parking lot. A total of 9 samples of beach sand were collected and transferred to Chulalongkorn University and samples for metabarcoding were kept at − 20 °C for further processing.

### ITS metabarcoding identification

#### Environmental DNA extraction and sequencing

Environmental DNA (eDNA) was extracted using the DNeasy PowerSoil Kit (Qiagen, USA) according to the manufacturer’s protocol. The extracted DNA was then used to amplify the fungal ITS1–2 barcode region using primers ITS1-F (5′-CTTGGTCATTTAGAGGAAGTAA-3′) and ITS2-R (5′-GCTGCGTTCTTCATCGATGC-3′)^61^ in combination with 2X sparQ HiFi PCR Master Mix (QuantaBio, USA). PCR consisted of an initial denaturation at 98 °C for 2 min, followed by 30 cycles of 98 °C for 20 s, 60 °C for 30 s, and 72 °C for 1 min, with a final extension at 72 °C for 1 min.

Amplicons were purified using sparQ PureMag Beads (QuantaBio, USA) and dual-indexed using 2.5 µL of each Nextera XT Index Primer (Illumina, San Diego, CA, USA) in a 50 µL reaction. The indexing PCR was performed with an initial denaturation at 98 °C for 2 min, followed by 10 cycles of 98 °C for 20 s, 60 °C for 30 s, and 72 °C for 30 s, and a final extension at 72 °C for 1 min. Indexed libraries were purified, pooled equimolarly, and diluted to a final loading concentration of 4 pM.

Negative control using PCR-grade water and positive control using laboratory stock fungal DNA were included to monitor contamination and amplification performance. Sequencing was conducted using 250 bp paired-end reads on an Illumina MiSeq v3 (Illumina, San Diego, CA, USA) at the OMICS Sciences and Bioinformatics Center, Faculty of Science, Chulalongkorn University, Thailand.

#### Bioinformatics process and analysis

The fungal amplicon sequences were processed using AMPtk: the Amplicon Toolkit for NGS data was used for analyzing the fungal ITS amplicon sequences following^62,63^, which demultiplexed ITS sequence range obtained from lower to higher (18,565 to 57,690 reads). Demultiplexed paired-end sequences were pre-processed into forward and reverse reads, truncated to a maximum of 300 bp; any read with a length less than 100 bp was eliminated. Using UNOISE3, the paired-end reads were combined to form a single long read where they overlapped. Chimeric ASVs were filtered using VSEARCH (v2.3.2)^64^, which excluded chimeras following a database comparison. Valid paired sequence readings were generated after pre-processing. The quality filtered reads were created by performing sequence quality filtering with an expected error parameter of 0.5% and yielded the amplicon sequence variants (ASVs)^65^. Finally, taxonomic assignment for ASVs was done using UNITE (v10.0/2024-04-21)^66^, and functional guilds were allocated using FUNGuild (v1.0)^67^.

All analyses and visualization were conducted in R v4.3.1^68^ using the phyloseq and microViz packages^69,70^. After filtering for fungal taxa, 614 ASVs from nine samples were retained. Fungal community composition, abundance, and diversity were analyzed. A Venn diagram of shared ASVs among beaches was created using the MicrobiotaProcess package^71^, and a bar plot of fungal genera was used to assess trophic modes based on the FUNGuild database^67^. Alpha diversity (observed ASVs) was compared among beach sites using two-way ANOVA with Tukey’s HSD test. Beta diversity was evaluated using Principal Coordinates Analysis (PCoA) based on Bray–Curtis dissimilarity. Principal Component Analysis (PCA) was performed on clr-transformed genus-level data using the default method. For Redundancy Analysis (RDA), clr-transformed data were filtered for taxa with ≥ 5% prevalence and constrained by moisture, pH, temperature, and sand type. Environmental effects were tested with PERMANOVA based on Aitchison distance (999 permutations). Opportunistic fungal pathogens, including WHO fungal priority pathogens and plant/animal pathogens, were identified based on WHO FPPL^3^ and FUNGuild, and visualized via a heatmap using the ComplexHeatmap package^72^.

### Culture-based identification

#### Isolation and culture condition

Fungal isolation was performed using TPS and SON samples that tested positive for WHO FPPL ASVs, as identified through ITS metabarcoding. Since the metabarcoding analysis required approximately three months to complete and given the nutrient-poor nature of beach sand, an enrichment cultivation approach was employed to enhance fungal viability and increase the likelihood of isolating target fungi. It was done by dissolving 1 g (w/v) of sand sample to 9 mL Sabouraud Dextrose Broth (SDB) and incubating at 25 °C and 35 °C for 3 days. One milliliter of SDB solution was spread on Sabouraud Dextrose Agar (SDA) and Malt Extract Agar (MEA) then incubated at the same condition for 7 to 14 days. The fungal characterization was done by observing the characteristics, macroscopic and microscopic (lactophenol blue wet mount) morphologies. The isolate was maintained in SDA slant for molecular identification and antifungal susceptibility testing.

#### Molecular identification

Genomic DNA (gDNA) was extracted from a fungus colony using a specially formulated lysis buffer consisting of 0.2 M NaCl, 0.02 M EDTA, 0.04 M Tris, 0.5% w/v SDS, 0.5% v/v β-mercaptoethanol, and 100 µg/ml proteinase K. The extraction process involved incubating the suspension at 65 °C for 3 h, followed by bead-beating for 3 min. Subsequently, the Maxwell^®^ RSC Whole Blood DNA Kit (Promega, Madison, USA) was used to purify the gDNA, which was then eluted in 40 µL of nuclease-free, molecular-grade water as modified from previously described protocol^73^. The full Internal Transcribed Spacer (ITS) region of the rRNA from all isolates was amplified using the primers ITS1 (5′-TCCGTAGGTGAACCTGCGG-3′) and ITS4 (5′-TCCTCCGCTTATTGATATGC-3′), as described previously^61^. For amplifying a partial section of the β-tubulin gene (exons 3–5 of tubC gene), the primer pairs of Bt2a (5′-GGTAACCAAATCGGTGCTGCTTTC-3′) and Bt2b (5′-ACCCTCAGTGTAGTGACCCTTGGC-3′) were used^74^. The PCR conditions for amplifying ITS were as follows: an initial denaturation step at 95 °C for 5 min, followed by 25 cycles consisting of 1 min at 95 °C, 25 s at 56 °C, and 30 s at 72 °C, with a final extension at 72 °C for 5 min. The PCR condition for amplifying β-tubulin gene were as same as ITS except the annealing steps of 30 s at 57 °C. For β-tubulin amplification, the conditions were identical to those used for ITS, except for the annealing step, which was performed at 57 °C for 30 s. ITS and β-tubulin amplicons were sequenced by Sanger sequencing then the forward and reverse sequences were assembled into contigs using DNABaser v5.20. Species identification was performed using BLASTn NCBI (blast.ncbi.nlm.nih.gov), based on query coverage and percent identity.

### Antifungal susceptibility test

For antifungal susceptibility testing (AFST), fungal cultures in the log phase on PDA at 35 °C were tested following the CLSI M38-A2 method^75,76^. In brief, spores of thirteen mold isolates were flushed by sterile 0.85% NaCl with 0.01% Tween 20 then the suspension adjusted with Sensititre™ YeastOne™ Broth (Cat. no. Y3462, Thermo Fisher scientific, Massachusetts, USA) to get the final concentration 10⁴ spores/mL. A 100 µL aliquot of each suspension was added to the wells of a Sensititre™ YeastOne™ AST Plate (Cat. no. YO10, Thermo Fisher Scientific, Waltham, Massachusetts, USA), which contained nine antifungal agents, echinocandins group: Amphotericin B (amp, 0.12–8 µg/mL), caspofungin (cas, 0.008–8 µg/mL), and micafungin (mic, 0.008–8 µg/mL); Azoles group: Fluconazole (flu, 0.12–256 µg/mL), itraconazole (itr, 0.015–16 µg/mL), voriconazole (vor, 0.008–8 µg/mL), anidulafungin (ani, 0.015–8 µg/mL), and posaconazole (pos, 0.008–8 µg/mL); and Pyrimidine analogs: 5-flucytosine (5-FC, 0.06–64 µg/mL). Antifungal susceptibility testing was performed using the Sensititre™ YeastOne™ YO10 system, a commercial colorimetric broth microdilution assay widely used in clinical laboratories and shown to have good agreement with CLSI reference methods for yeasts and *Aspergillus* spp^56,57^. However, as this system is primarily optimized for yeasts, results for filamentous fungi should be interpreted as screening or indicative rather than definitive susceptibility measurements. MIC endpoints were determined based on inhibition of fungal growth according to the manufacturer’s instructions and CLSI M38-based principles for molds^76^. Due to the absence of established clinical breakpoints or epidemiological cutoff values (ECOFFs) for several mold–drug combinations, MIC values were interpreted descriptively, and isolates were not categorized as susceptible or resistant.

### Intraspecific relation of antifungal resistance isolate

The phylogeography of the most antifungal-resistant isolate, *Aspergillus terreus*, was investigated by analyzing gene flow using haplotypes derived from the ITS region and β-tubulin sequences. Sequences were aligned based on the *Aspergillus* sp. section *Terrei* following Visagie et al., (2024)^77^, and trimmed using MEGA11 software (v11.0.13). *Aspergillus terreus* strains TPS1-1 and TPS1-2 were analyzed alongside comparative strains based on environmental and clinical sources (*n* = 54, Table S4), including *A. terreus* (Thailand, environmental [2]; India, clinical [1]; Malaysia, environmental [7]; Spain, clinical [10]), *A. alabamensis* (United States, clinical/environmental [5]; Argentina, environmental [2]), *A. pseudoalabamensis* (United States, clinical [3]), *A. citrinoterreus* (United States, clinical [1]; Spain, clinical [17]), and *A. hortae* (United States, environmental [2]; Ecuador, environmental [1]; Brazil, clinical [1]). *A. neoniveus* section Flavipedes was used as the outgroup^58,77–83^. Each gene barcode was constructed to get the phylogenetic tree using maximum likelihood (ML) by IQ-TREE 2 v2.4.0^84^. ML tree was constructed using the ITS region (477 bp) and β-tubulin (467 bp), with the best-fit models TN + F and K2P+G4, respectively, selected based on the Bayesian Information Criterion (BIC). To improve phylogenetic resolution by incorporating both variable and conserved regions, the ITS and β-tubulin sequences were concatenated after excluding missing data, ambiguous nucleotides, and alignment gaps, resulting in a final alignment of 792 bp. This concatenated dataset was then used to construct a consensus ML tree using the K2P+G4 model, as determined by BIC. Tree inference was performed using a nearest-neighbor interchange (NNI) search on the 20 best initial trees to optimize the topology. Branch support values were assessed through 1,000 ultrafast bootstrap replicates and 1,000 likelihood ratio tests to ensure statistical robustness. To understand the intraspecific of clinical and environmental strain of *A. terreus* strain TPS1-1 and TPS1-2, haplotype was performed by preparing the haplotype sequence data using DnaSP (Dna Sequence Polymorphism) v6.12.03, then analyzed and visualized with PopART v1.7 using TCS network^85^ following the haplotype traits data (Table S5).

## Supplementary Information

Below is the link to the electronic supplementary material.

Supplementary Material 1

## Data Availability

Raw sequence reads generated in this study have been deposited in the National Center for Biotechnology Information (NCBI) Sequence Read Archive (SRA) under BioProject accession number PRJNA1273000. All culture-derived isolate sequences are publicly available in the NCBI GenBank database. ITS sequences were deposited under accession numbers PV639331, PV639332, and PV762228–PV762242, while β-tubulin sequences were deposited under accession numbers PV755998–PV756014.The deposited sequences correspond to the following isolates: *Aspergillus* section *Terrei* TPS1-1 (ITS: PV639331; β-tubulin: PV755998), *Aspergillus* section *Terrei* TPS1-2 (PV639332; PV755999), *Acrophialophora levis* TPS3-3 (PV762228; PV756000), *Aspergillus* sp. TPS3-4 (PV762229; PV756001), *Aspergillus laciniosus* TPS3-5 (PV762230; PV756002), *Corynascella* sp. TPS2-6 (PV762231; PV756003), *Caliciopsis* sp. SON3-8 (PV762233; PV756005), *Fusarium neocosmosporiellum* TPS1-10 (PV762235; PV756007), *Trichoderma *sp. TPS1-11 (PV762236; PV756008), *Aspergillus tubingensis* TPS1-12 (PV762237; PV756009), *Montagnula* sp. SON3-13 (PV762238; PV756010), *Trichoderma* sp. TPS2-15 (PV762240; PV756012), and *Montagnula* sp. SON3-17 (PV762242; PV756014).
