# Supplementary material for: Opportunistic fungi in tropical beaches: Diversity, WHO fungal priority pathogens, and antifungal resistance case study
Source: Sci Rep. 2026 Jun 4;16:26459. doi: 10.1038/s41598-026-55443-7 (PMC13500499; doi:10.1038/s41598-026-55443-7)
Supplement: Supplementary file 1 [file 41598_2026_55443_MOESM1_ESM.docx]

**– Supplementary Data –**

**Opportunistic Fungi in Tropical Beaches: Diversity, WHO Fungal Priority Pathogens, and Antifungal Resistance Case Study**

Syahriar Nur Maulana Malik Ibrahim^1,2^, Chayaporn Lakmuang^1,3^, Teeratat Kaewjon^1^, Panwad Tongchai^4^, Gregorius Nico Adi Setiawan^1^, Phetcharin Hinmo^1^, Ariya Chindamporn^2,4^, Nattapol Kraisitudomsook^2,5^, Nuttapon Pombubpa^1,2*^

*1 – Department of Microbiology, Faculty of Science, Chulalongkorn University, Bangkok 10330, Thailand*

*2 – Southeast Asia Antifungal Resistance Monitoring Initiative (SEA-ARMi), Department of Microbiology, Faculty of Science, Chulalongkorn University, Bangkok 10330, Thailand*

*3 – Bioinformatics and Computational Biology Program, Graduate School, Chulalongkorn University, Bangkok 10330, Thailand*

*4 - Department of Microbiology, Faculty of Medicine, Chulalongkorn University, Bangkok 10330, Thailand*

*5 - Department of Microbiology, Faculty of Science, Mahidol University, Bangkok 10400, Thailand*

*Corresponding author, Email: [Nuttapon.Po@chula.ac.th](mailto:Nuttapon.Po@chula.ac.th)

**Figure S1**. Fungal community diversity and composition across beach sites. Beta diversity is shown by (**a**) PCA and (**b**) PCoA, colored by sampling site: Thap Sakae (red), Don Samran (green), and Son (blue). PCA was performed on clr-transformed genus-level data, with vectors representing the top 10 contributing genera. PCoA based on Bray–Curtis dissimilarity showed significant differences in community composition among sites (PERMANOVA, R² = 0.383, p = 0.005). (**c**) Homogeneity of multivariate dispersion among beach sites. Boxplots show distances to group centroids for each beach (Thap Sakae, Don Samran, and Son) based on Bray–Curtis dissimilarity. Dispersion differed significantly among sites (betadisper, F = 11.33, *p* = 0.037).

**Table S1.** Fifty-eight opportunistic fungal pathogens based on the FUNGuild database.

| **Animal opportunistic pathogen** | | | |
| --- | --- | --- | --- |
| **No.** | **Species** | **guild** | **ASVs** |
| 1. | *Beauveria* sp*.* | Animal Pathogen | ASV825 |
| 2. | *Biatriospora* sp. | Animal Pathogen-Endophyte-Wood Saprotroph | ASV559 |
| 3. | *Candida orthopsilosis* | Animal Pathogen | ASV124 |
| 4. | *Candida theae* | Animal Pathogen-Endophyte-Endosymbiont-Epiphyte-Soil Saprotroph-Undefined Saprotroph | ASV148 |
| 5. | *Candida* sp. | Animal Pathogen-Endophyte-Endosymbiont-Epiphyte-Soil Saprotroph-Undefined Saprotroph | ASV288 |
| 6. | *Cyphellophora europaea* | Animal Pathogen | ASV65 |
| 7. | *Exophiala nishimurae* | Animal Pathogen-Fungal Parasite-Undefined Saprotroph | ASV668 |
| 8. | *Exophiala dermatitidis* | Animal Pathogen-Undefined Saprotroph | ASV218 |
| 9. | *Meyerozyma guilliermondii* | Animal Pathogen | ASV60 |
| 10. | *Rasamsonia eburnea* | Animal Pathogen | ASV476 |
| 11. | *Scedosporium boydii* | Animal Pathogen | ASV407 |
| 12. | *Talaromyces aurantiacus* | Animal Pathogen | ASV62 |
| 13. | *Wickerhamomyces anomalus* | Animal Pathogen | ASV593 |
| **Plant opportunistic pathogen** | | | |
| **No.** | **Species** | **guild** | **ASVs** |
| 1. | *Apiospora pseudospegazzinii* | Plant Pathogen | ASV670 |
| 2. | *Aplosporella* sp*.* | Plant Pathogen | ASV191 |
| 3. | *Aspergillus aculeatus* | Plant Pathogen | ASV69 |
| 4. | *Cochliobolus akaiiensis* | Plant Pathogen | ASV438 |
| 5. | *Curvularia sp.* | Plant Pathogen | ASV565 |
| 6. | *Edenia gomezpompae* | Plant Pathogen | ASV894 |
| 7. | *Entorrhiza cypericola* | Plant Pathogen | ASV419 |
| 8. | *Eutypa sp.* | Plant Pathogen | ASV685 |
| 9. | *Eutypella* sp*.* | Plant Pathogen | ASV310 |
| 10. | *Ganoderma nasalaense* | Plant Pathogen-Wood Saprotroph | ASV145 |
| 11. | *Ganoderma gibbosum* | Plant Pathogen-Wood Saprotroph | ASV268 |
| 12. | *Ganoderma* sp*.* | Plant Pathogen-Wood Saprotroph | ASV348 |
| 13. | *Itersonilia perplexans* | Litter Saprotroph-Plant Pathogen | ASV82 |
| 14. | *Lasiodiplodia* sp*.* | Plant Pathogen | ASV566 |
| 15. | *Lasiodiplodia acaciae* | Plant Pathogen | ASV717 |
| 16. | *Letendraea* sp*.* | Endophyte-Lichen Parasite-Plant Pathogen-Undefined Saprotroph | ASV15 |
| 17. | *Marasmius haematocephalus* | Endophyte-Plant Pathogen-Undefined Saprotroph | ASV335 |
| 18. | *Massarina* sp*.* | Plant Pathogen-Undefined Saprotroph | ASV318 |
| 19. | *Neodevriesia coccolobae* | Plant Pathogen-Undefined Saprotroph | ASV327 |
| 20. | *Neodevriesia strelitziicola* | Plant Pathogen-Undefined Saprotroph | ASV897 |
| 21. | *Neokalmusia didymospora* | Endophyte-Lichen Parasite-Plant Pathogen-Undefined Saprotroph | ASV447 |
| 22. | *Nigrospora oryzae* | Endophyte-Plant Pathogen | ASV126 |
| 23. | *Peniophora taiwanensis* | Plant Pathogen-Wood Saprotroph | ASV6 |
| 24. | *Peniophora* sp*.* | Plant Pathogen-Wood Saprotroph | ASV70 |
| 25. | *Periconia thailandica* | Endophyte-Plant Pathogen-Wood Saprotroph | ASV170 |
| 26. | *Periconia epilithographicola* | Endophyte-Plant Pathogen-Wood Saprotroph | ASV181 |
| 27. | *Periconia cookei* | Endophyte-Plant Pathogen-Wood Saprotroph | ASV855 |
| 28. | *Peroneutypa* sp*.* | Plant Pathogen | ASV10 |
| 29. | *Pestalotiopsis* sp*.* | Plant Pathogen | ASV770 |
| 30. | *Phaeophleospora hymenocallidicola* | Plant Pathogen | ASV681 |
| 31. | *Phellinus fastuosus* | Plant Pathogen-Wood Saprotroph | ASV671 |
| 32. | *Phoma* sp*.* | Endophyte-Dung Saprotroph-Lichen Parasite-Litter Saprotroph-Plant Pathogen-Soil Saprotroph-Wood Saprotroph | ASV437 |
| 33. | *Pseudopithomyces* sp*.* | Endophyte-Lichen Parasite-Plant Pathogen-Undefined Saprotroph | ASV8 |
| 34. | *Psilocybe cubensis* | Dung Saprotroph-Plant Parasite-Soil Saprotroph-Undefined Saprotroph-Wood Saprotroph | ASV37 |
| 35. | *Pyrenochaeta* sp*.* | Plant Pathogen-Wood Saprotroph | ASV739 |
| 36. | *Xenocamarosporium acaciae* | Endophyte-Lichen Parasite-Plant Pathogen-Undefined Saprotroph | ASV150 |
| **Animal and plant opportunistic pathogen** | | | |
| **No.** | **Species** | **guild** | **ASVs** |
| 1. | *Acremonium pinkertoniae* | Animal Pathogen-Endophyte-Fungal Parasite-Plant Pathogen-Wood Saprotroph | ASV605 |
| 2. | *Acremonium polychromum* | Animal Pathogen-Endophyte-Fungal Parasite-Plant Pathogen-Wood Saprotroph | ASV636 |
| 3. | *Fusarium* sp*.* | Animal Pathogen-Endophyte-Lichen Parasite-Plant Pathogen-Soil Saprotroph-Wood Saprotroph | ASV110 |
| 4. | *Fusicolla violacea* | Animal Pathogen-Endophyte-Fungal Parasite-Lichen Parasite-Plant Pathogen-Wood Saprotroph | ASV107 |
| 5. | *Paracremonium* sp*.* | Animal Pathogen-Endophyte-Fungal Parasite-Lichen Parasite-Plant Pathogen-Wood Saprotroph | ASV708 |
| 6. | *Teratosphaeria* sp*.* | Animal Pathogen-Plant Pathogen-Undefined Saprotroph | ASV541 |
| 7. | *Trichoderma* sp*.* | Animal Pathogen-Endophyte-Epiphyte-Fungal Parasite-Plant Pathogen-Wood Saprotroph | ASV173 |
| 8. | *Trichoderma reesei* | Animal Pathogen-Endophyte-Epiphyte-Fungal Parasite-Plant Pathogen-Wood Saprotroph | ASV209 |
| 9. | *Wallrothiella* sp*.* | Animal Pathogen-Endophyte-Fungal Parasite-Plant Pathogen-Wood Saprotroph | ASV702 |

**Table S2.** The comparison results from identification based on culture-based identification (BLAST NCBI) to ITS metabarcoding identification.

| **Isolate no.** | **Sampling Site** | **ITS identification** | | | | **Beta-tubulin identification** | | | | **Culture-based identification**  **(Accession no.)** | **ITS metabarcoding identifcation (existence)** | **Match-making result** |
| --- | --- | --- | --- | --- | --- | --- | --- | --- | --- | --- | --- | --- |
|  |  | Query cover | Percent identity | Species | Isolated source | Query cover | Percent Identity | Species | Isolated source |  |  |  |
| 1 | TPS1 | 99% | 97% | *Aspergillus terreus* isolate DC-17 (OK465110.1) | marine mud | 100% | 99.63% | *Aspergillus* *terreus* (KM491899.1) | surface water | ***Aspergillus*** ***terreus* TPS1-1**  ITS (PV639331)  Beta-tubulin (PV755998) | Positive (exist) | **Match** (genus) |
| 2 | TPS1 | 99% | 97% | *Aspergillus* *terreus* (KR704571.1) | host Fimbristylis ferruginea | 100% | 96.85% | *Aspergillus* *terreus* isolate A1S2_D18 (JX501417.1) | NA | ***Aspergillus* *terreus* TPS1-2**  ITS (PV639332)  Beta-tubulin (PV755999) | Positive (exist) | **Match** (genus) |
| 3 | TPS1 | 99% | 92% | *Acrophialophora* *levis* isolate lung (MN461541.1) | human lung | 100% | 82.66% | *Acrophialophora* *levis* strain UTHSC DI-13-150 (LN624432.1) | soil | ***Acrophialophora* *levis* TPS3-3**^a^  ITS (PV762228)  Beta-tubulin (PV756000) | **Negative** (not exist) | **Mismatch** (negative in metabarcoding result)^a^ |
| 4 | TPS3 | 100% | 100% | *Aspergillus* *fischeri* strain CBS:101.12 (MH854602.1) | NA | 100% | 98.52% | *Aspergillus* *laciniosus* voucher BAFC:4644 (MH445504.1) | NA | ***Aspergillus*** **sp. isolate TPS3-4**  ITS (PV762229)  Beta-tubulin (PV756001) | Positive (exist) | **Match** (genus) |
| 5 | TPS3 | 100% | 99.58% | *Aspergillus* *laciniosus* strain SM12 (MH595814.1) | NA | 100% | 98.51% | *Aspergillus* *laciniosus* voucher BAFC:4644 (MH445504.1) | NA | ***Aspergillus* *laciniosus* TPS3-5**  ITS (PV762230)  Beta-tubulin (PV756002) | Positive (exist) | **Match** (genus) |
| 6 | TPS2 | 100% | 87.73%***** | *Corynascella* sp. strain BY6 (MZ380046.1) | NA | 98% | 79.24%***** | *Schizothecium* *fimbriatum* strain CBS 144.54 (AY999156.1) | NA | ***Corynascella* sp. TPS2-6**^a^  ITS (PV762231)  Beta-tubulin (PV756003) | **Negative** (not exist) | **Mismatch** (negative in metabarcoding result)^a^ |
| 7 | SON3 | 38% | 88.44%***** | *Nothomicrothyrium* *beaucarneae* strain CBS:150084 (OR680761.1) | NA | 82% | 83.02%***** | *Caliciopsis* *moriondi* isolate 4 (MN150123.1) | NA | ***Caliciopsis* sp. SON3-8**^a^  ITS (PV762233)  Beta-tubulin (PV756005) | **Negative** (not exist) | **Mismatch** (negative in metabarcoding result)^a^ |
| 8 | TPS1 | 100% | 99.41% | *Fusarium* *neocosmosporiellum* strain R1 (OQ860749.1) | NA | 83% | 99.11% | *Fusarium* *neocosmosporiellum* isolate FN9 (OK180434.1) | peanuts | ***Fusarium* *neocosmosporiellum* TPS1-10**^b^  ITS (PV762235)  Beta-tubulin (PV756007) | **Positive** (exist but not the same correct site) | **Mismatch** (negative in TPSsoil1, positive in SONsoil3)^b^ |
| 9 | TPS1 | 100% | 100% | *Trichoderma* *harzianum* strain NECC30420 (MH153618.1) | soil under Abies fabri | 100% | 95.80% | *Trichoderma* sp. strain RHP-2022a (ON238008.1) | decaying stem | ***Trichoderma* sp. TPS1-11**  ITS (PV762236)  Beta-tubulin (PV756008) | Positive (exist) | **Match** (genus) |
| 10 | TPS1 | 100% | 100% | *Aspergillus* *tubingensis* isolate tissue (OR838476.1) | NA | 100% | 100% | *Aspergillus* *tubingensis* isolate A3S3_6 (JX545082.1) | NA | ***Aspergillus* tubingensis TPS1-12**  ITS (PV762237)  Beta-tubulin (PV756009) | Positive (exist) | **Match** (genus) |
| 11 | SON3 | 100% | 99.44% | *Montagnula* *scabiosae* strain 2-F29 (MW081285.1) | sponge | 91% | 87.64%***** | *Tremateia* sp. SAMC170 (MN481606.1) | dead branch | ***Montagnula* sp. SON3-13**  ITS (PV762238)  Beta-tubulin (PV756010) | Positive (exist) | **Match** (genus) |
| 12 | TPS2 | 100% | 100% | *Trichoderma* sp. strain BA132 (MK613172.1) | soil | 100% | 95.93% | *Trichoderma* sp. RHP-2022a (ON238008.1) | decaying bamboo stem | ***Trichoderma* sp. TPS2-15**  ITS (PV762240)  Beta-tubulin (PV756012) | Positive (exist) | **Match** (genus) |
| 13 | SON3 | 100% | 99.62% | *Montagnula* *scabiosae* isolate PKMS 13.1 (MZ502216.1) | sponge | 91% | 87.64%***** | *Tremateia* sp. SAMC170 (MN481606.1) | dead branch | ***Montagnula* sp. SON3-17**  ITS (PV762242)  Beta-tubulin (PV756014) | Positive (exist) | **Match** (genus) |

Asterisk (*) indicates sequence identity <90%

^a^ No corresponding sequence detected in the ITS metabarcoding dataset.

^b^ Sequence detected in the metabarcoding dataset but assigned to a different sampling site.

**Table S3.** Geophysicochemical condition of sampling sites.

| **Beach location** | **Geographical site** | | | **Sample conditions** | | | |
| --- | --- | --- | --- | --- | --- | --- | --- |
|  | **Area condition** | **Coordinate** | **Elevation (m)** | **Moisture** | **Sand types** | **pH** | **Temperature (°C)** |
| Thap Sakae | Within 500 m of housing | 11.495,  99.632 | 0 | 8 | Coarse | 6 | 35.5 |
| Don Samran | >1 km of housing | 11.277,  99.550 | 20 | 4.5 | Medium | 5 | 30.5 |
| Son | Within 500 m of housing | 11.096,  99.490 | 10 | 6.6 | Medium | 5 | 32.3 |

**Table S4.** The ITS and β-Tubulin sequences of *Aspergillus terreus* in China, India, Malaysia, Spain, USA, Argentina, Ecuador, Brazil and Thailand for phylogenetic and haplotype analysis. An outgroup that used for phylogenetic construction was *Aspergillus neoniveus* (subgen*. Circumdati* sect*. Flavipedes*).

| **ITS** | **β-Tubulin** | **Strains** | **Source** | **Location** | **Reference** |
| --- | --- | --- | --- | --- | --- |
| PV639331 | PV755998 | *Aspergillus terreus* isolate TPS1-1 | Sand beach sample | Thap Sakae Beach, Prachuap Khiri Khan Province, Thailand | This study |
| PV639332 | PV755999 | *A. terreus* isolate TPS1*-2* |  |  |  |
| MT703807.1 | MT929185.1 | *A. terreus* strain AG499 | Organic fertilizer | Tha Makham Natural Farming Center, Kanchanaburi Province, Thailand | Arnthong et al. (2020)^58^ |
| MT703802.1 | MT929181.1 | *A. terreus* strain AG438 |  |  |  |
| KX792117.1 | KX879757.1 | *A. terreus* strain NFCCI 3998 | Corneal scraping | India | Unpublsihed |
| KF669498.1 | KF669500.1 | *A. terreus* strain USMR11 | Corn feed | Malaysia | Unpublsihed |
| KF669506.1 | KF669507.1 | *A. terreus* strain USMA10 | Unknown |  |  |
| MT936887.1 | MT955678.1 | *A. terreus* strain PN3 | rubber seed |  |  |
| JX501389.1 | JX501421.1 | *A. terreus* strain A2S4_D7 | Sand beach sample | Batu Ferringhi beach, Penang Island, Malaysia | Teh & Latiffah (2015)^79^ |
| JX501372.1 | JX501419.1 | *A. terreus* strain A2S1_D106 |  |  |  |
| JX501353.1 | JX501417.1 | *A. terreus* strain A1S2_D18 |  |  |  |
| JX501402.1 | JX501422.1 | *A. terreus* strain A3S5_1 |  |  |  |
| KP175286.1 | LN680675 | *A terreus* strain GM1738 | Human patient | Madrid, Spain. | Guienna et al. (2015)^82^ |
| KP175287.1 | LN680676 | *A. terreus* strain GM1832 |  |  |  |
| KP175288.1 | LN680677 | *A. terreus* strain GM1839 |  |  |  |
| KP175289.1 | LN680678 | *A. terreus* strain GM1876 |  |  |  |
| KP175290.1 | LN680679 | *A. terreus* strain GM2001 |  |  |  |
| KP175291.1 | LN680680 | *A. terreus* strain GM2036 |  |  |  |
| KP175292.1 | LN680681 | *A. terreus* strain GM2042 |  |  |  |
| KP175293.1 | LN680682 | *A. terreus* strain GM2088 |  |  |  |
| KP175294.1 | LN680683 | *A. terreus* strain GM2186 |  |  |  |
| KP175295.1 | LN680684 | *A. terreus* strain GM2263 |  |  |  |
| FJ531193 | FJ491731 | *Aspergillus alabamensis* strain UAB 20 | Wound | Alabama, USA | Samson et al. (2011)^80^; Zhang et al. (2018)^78^ |
|  |  | *A. alabamensis* strain UAB 15 | Sputum | Alabama, USA | Samson et al. (2011)^80^ |
|  |  | *A. alabamensis* strain NRRL 29810 = CBS 125693 | Soil | Florida, USA |  |
|  |  | *A. alabamensis* strain DTO 15-F8 | Soil | Argentina |  |
|  |  | *A. alabamensis* strain DTO 15-F9 | Soil | Argentina |  |
| OL765286.1 | OL792693.1 | *Aspergillus pseudoalabamensis* strain UTHSCSA DI21-178 | Human patient | Colorado, USA | Cañete-Gibas et al. (2023)^83^ |
| OL765283 | OL792695 | *A. pseudoalabamensis* strain UTHSCSA DI16-487 | Human patient, right ethmoid sinus |  |  |
| OL765284 | OL792696 | *A. pseudoalabamensis* strain UTHSCSA DI16-488 | Human patient, ear swab |  |  |
| EF669579 | EF669518 | *Aspergillus citrinoterreus* strain NRRL 1913 | Lung of pocket mouse | Arizona, USA | Zhang et al. (2018)^78^; Peterson (2008)^81^ |
| KP175260 | LN680657 | *A. citrinoterreus* strain GM228 | Sputum | Madrid, Spain. | Guienna et al. (2015)^82^; Zhang et al. (2018)^78^ |
| KP175261 | LN680658 | *A.* citrinoterreus strain GM464 | Bronchial secretion |  |  |
| KP175262 | LN680659 | *A. citrinoterreus* strain GM541 | Human patient | Madrid, Spain. | Guienna et al. (2015)^82^ |
| KP175263 | LN680660 | *A. citrinoterreus* strain GM673 |  |  |  |
| KP175264 | LN680661 | *A. citrinoterreus* strain GM676 |  |  |  |
| KP175265 | LN680662 | *A. citrinoterreus* strain GM837 |  |  |  |
| KP175266 | LN680663 | *A. citrinoterreus* strain GM1381 |  |  |  |
| KP175270 | LN680664 | *A. citrinoterreus* strain GM1532 |  |  |  |
| KP175271 | LN680665 | *A. citrinoterreus* strain GM1597 |  |  |  |
| KP175273 | LN680667 | *A. citrinoterreus* strain GM2006 |  |  |  |
| KP175274 | LN680668 | *A. citrinoterreus* strain GM2025 |  |  |  |
| KP175275 | LN680669 | *A. citrinoterreus* strain GM2137 |  |  |  |
| KP175276 | LN680670 | *A. citrinoterreus* strain GM2568 |  |  |  |
| KP175277 | LN680671 | *A. citrinoterreus* strain GM2597 |  |  |  |
| KP175278 | LN680672 | *A. citrinoterreus* strain GM3921 |  |  |  |
| KP175284 | LN680673 | *A. citrinoterreus* strain GM4511 |  |  |  |
| KP175285 | LN680674 | *A. citrinoterreus* strain GM4611 |  |  |  |
| FJ531209 | FJ491711 | *Aspergillus hortae* strain CBS 124230 | Clinical isolate | Brazil | Zhang et al. (2018)^78^; Samson et al. (2011)^80^ |
|  |  | *A. hortae* strain IBT 16744 | Soil | Galapagos Islands, Ecuador | Samson et al. (2011)^80^ |
|  |  | *A. hortae* strain IBT 16745 | Soil | Florida, USA | Samson et al. (2011)^80^ |
|  |  | *A. hortae* strain IBT 6271 | Soil | Florida, USA | Samson et al. (2011)^80^ |
| EF669612.1 | EU014098.1 | *Aspergillus neoniveus* strain NRRL 5299 | Outgroup (subgen*. Circumdati* sect*. Flavipedes*) | Arizona,  USA | Peterson (2008)^81^ |
| FJ531198.1 | FJ491722.1 | *A. neoniveus* strain CBS 261.73 |  | Forest soil, Thailand | Zhang et al. (2018)^78^; Visagie et al. (2024)^77^ |

**Table S5.** Haplotype analysis results from 52 *Aspergillus* spp. section *Terrei* of ITS and β-tubulin concatenation sequence (792 bp) based on the country source.

| **Haplotype** | **Country** | | | | | | | |
| --- | --- | --- | --- | --- | --- | --- | --- | --- |
|  | **Thailand** | **India** | **Malaysia** | **Argentina** | **USA** | **Spain** | **Brazil** | **Ecuador** |
| **Hap_1** | 2 | 0 | 0 | 0 | 1 | 0 | 0 | 0 |
| **Hap_2** | 1 | 1 | 2 | 0 | 0 | 0 | 0 | 0 |
| **Hap_3** | 0 | 0 | 1 | 0 | 0 | 0 | 0 | 0 |
| **Hap_4** | 0 | 0 | 0 | 0 | 0 | 10 | 0 | 0 |
| **Hap_5** | 1 | 0 | 0 | 0 | 0 | 0 | 0 | 0 |
| **Hap_6** | 0 | 0 | 1 | 0 | 0 | 0 | 0 | 0 |
| **Hap_7** | 0 | 0 | 1 | 0 | 0 | 0 | 0 | 0 |
| **Hap_8** | 0 | 0 | 1 | 0 | 0 | 0 | 0 | 0 |
| **Hap_9** | 0 | 0 | 1 | 0 | 0 | 0 | 0 | 0 |
| **Hap_10** | 0 | 0 | 0 | 0 | 1 | 0 | 0 | 0 |
| **Hap_11** | 0 | 0 | 0 | 0 | 2 | 0 | 0 | 0 |
| **Hap_12** | 0 | 0 | 0 | 0 | 1 | 0 | 1 | 1 |
| **Hap_13** | 0 | 0 | 0 | 0 | 1 | 16 | 0 | 0 |
| **Hap_14** | 0 | 0 | 0 | 0 | 0 | 1 | 0 | 0 |
| **Hap_15** | 0 | 0 | 0 | 0 | 1 | 0 | 0 | 0 |
| **Hap_16** | 0 | 0 | 0 | 1 | 0 | 0 | 0 | 0 |
| **Hap_17** | 0 | 0 | 0 | 1 | 0 | 0 | 0 | 0 |
| **Hap_18** | 0 | 0 | 0 | 0 | 2 | 0 | 0 | 0 |
